# Enhancing Fresh-Cut Apple Preservation: Impact of Slightly Acidic Electrolyzed Water and Chitosan–Apple Essence Microencapsulation Coating on Browning and Flavor

**DOI:** 10.3390/foods13101585

**Published:** 2024-05-20

**Authors:** Zhenyu Luo, Guijing Li, Yanlin Du, Junjie Yi, Xiaosong Hu, Yongli Jiang

**Affiliations:** 1 Faculty of Food Science and Engineering, Kunming University of Science and Technology, Kunming 650500, China; lzy15708797717@163.com (Z.L.); m13238708120@163.com (G.L.); duyanlin_work@163.com (Y.D.); junjieyi@kust.edu.cn (J.Y.); huxiaos@263.net (X.H.); 2 Key Laboratory of Plateau Characteristic Prepared Food in Yunnan Province, Kunming 650500, China; 3 Yunnan Engineering Research Center for Fruit & Vegetable Products, Kunming 650500, China; 4 College of Food Science and Nutritional Engineering, China Agricultural University, Beijing 100083, China

**Keywords:** fresh-cut apple, apple essence microencapsulation, chitosan coating, browning, flavor

## Abstract

Fresh-cut apple preservation is a critical concern in the food industry due to the rapid deterioration of texture, color, and flavor. While our previous study introduced apple essence microencapsulation (AEM) to enhance flavor during storage, its impact on overall storage quality was minimal. Thus, this study explores the application of two preservation techniques, namely, slightly acidic electrolyzed water (SAEW) and chitosan–apple essence microencapsulation (CH–AEM) coating, to enhance the quality of fresh-cut apples. Our findings reveal that SAEW treatment significantly reduces the browning index (from 65.38 to 57.36) and respiratory rate (from 5.10% to 4.30% of CO_2_), and maintains a desirable aroma profile compared to uncoated treatment during 10 days of storage. Additionally, the CH–AEM coating acts as a protective barrier, further preserving the sensory characteristics of fresh-cut apples. Notably, the SAEW–CH–AEM group exhibits superior performance in firmness (8.14 N), respiratory rate (3.37% of CO_2_), ion leakage (34.86%), and juice yield (47.52%) after 10 days. Our research highlights the synergistic effect of combining these preservation strategies, providing a promising approach for extending the shelf life of fresh-cut apples while maintaining their visual appeal and aromatic quality. These results offer valuable insights for the fresh-cut produce industry, contributing to improved apple product preservation and consumer satisfaction.

## 1. Introduction

With the fast-paced lifestyle prevalent today, fresh-cut fruits are gaining traction among consumers, emerging as a primary choice for fruit consumption owing to their freshness, health benefits, cleanliness, and convenience [1,2]. Among these, fresh-cut apples stand out as a globally cherished fruit, renowned for their distinct flavor [3,4]. Nevertheless, akin to other fresh-cut fruits and vegetables, the minimal processing involved in fresh-cut apples makes them susceptible to quality deterioration, including water loss, softening, enzyme browning, microbial contamination, and aroma loss [5,6]. Consequently, extending the shelf life of fresh-cut apples while upholding product quality has become a focal point in contemporary research.

Quality control of fresh-cut products hinges on preserving aroma [7,8]. In a previous study, we formulated an apple essence microencapsulation (AEM) using β-cyclodextrin, gum arabic, and montmorillonite via Plackett–Burman and Box–Behnken designs. Application of the optimized AEM in fresh-cut apple preservation improved flavor during storage but had minimal impact on overall storage quality (data not yet published). Therefore, other preservation techniques must be combined to maintain the postharvest quality of fresh-cut apples. Chitosan (CH) is one of the most promising materials due to its nontoxicity, favorable oxygen barrier ability, biocompatibility, and antimicrobial activity [3,9]. Numerous studies have shown that CH coating holds significant potential for application in fresh-cut apple preservation [3,6,9,10]. Therefore, AEM can be considered to be added to CH to synergistically enhance the freshness of fresh-cut apples. However, there is a scarcity of reports on related research in this area.

Apart from aroma loss, browning is another challenge for fresh-cut apple preservation, which significantly affects consumer perceptions among other sensory parameters [11,12]. Various methods have been utilized to combat browning in fresh-cut fruits and vegetables, including thermal treatment, low temperature storage, shortwave ultraviolet, high-pressure processing, dense phase carbon dioxide treatment, pulsed electric field processing, ultrasound treatment, and more [9,12,13]. One such method gaining attention is slightly acidic electrolyzed water (SAEW) with a pH range of 5.0–6.5. SAEW is favored for its eco-friendliness, cost-effectiveness, and ease of application in food preservation. It has been widely adopted as a browning inhibitor in various fresh-cut fruits and vegetables, such as kiwifruit [14], bell peppers [15], and others. Studies have shown promising results regarding SAEW’s efficacy as an anti-browning agent for fresh-cut apples [16]. Furthermore, SAEW has been found to possess additional functions, such as inhibiting microbial growth on the surface of apple slices, preserving their high nutritional value and firm texture, and minimizing weight loss [17]. Currently, research on preservation primarily centers on individual treatments such as single CH coating or SAEW treatment, with limited exploration of synergistic applications. Generally, single preservation techniques may struggle to achieve the desired effects; thus, combining multiple methods holds greater promise for effective preservation.

Consequently, the aim of this study was to evaluate the impact of CH–AEM coating in conjunction with SAEW treatments on fresh-cut apple preservation. To the best of our knowledge, there is no existing literature on the combined application of CH–AEM and SAEW treatment for food preservation. This research endeavors to provide novel insights into mitigating browning and aroma loss in fresh-cut apples during storage, presenting innovative approaches for the preservation of fresh-cut fruits and vegetables.

## 2. Materials and Methods

### 2.1. Materials and Reagents

The experiment utilized “Red Fuji” apples, sourced from local orchards in Zhaotong, China. These apples were selected based on uniform maturity levels and were free from pests, diseases, or mechanical damage. SAEW with an available chlorine concentration of 30 mg/L and pH 6.0 was produced using the “SOLXN” SAEW generator (SW-G20, Zhejiang SOLXN Technology Co., Ltd., Hangzhou, China) and electrolyzing a saturated sodium chloride solution. CH was procured from Shandong AK Biotech in Jinan, China. Apple essence was supplied by Shangqiu Qingshan Spices Co., Ltd. in Shangqiu, China. β-cyclodextrin and montmorillonite were obtained from Yuanye BioTechnology Co., Ltd. in Shanghai, China. Gum arabic (GA) and other chemicals and reagents were of analytical grade and were sourced from Aladdin Reagent Co., Ltd. in Shanghai, China.

### 2.2. CH–AEM Coating Solution Preparation

AEM was prepared using an ultrasonic homogenization method following the procedures of Li et al. [18] with some modifications. Briefly, the experimental procedure involved preparing a saturated solution of β-CD by mixing an appropriate amount with 100 mL of preheated distilled water in a 250 mL three-necked flask. Subsequently, varying amounts of GA and MMT were dissolved together in the β-CD saturated solution and heated in a water bath (THM-2865, Thermo Fisher Scientific, Beijing, China) at 50 °C for 20 min. Following this, different quantities of apple essence were added to the solution described above. Subsequently, the initial suspension underwent a process of ultrasonic treatment at 100 W, 50 °C for 2 min, utilizing an ultrasonication bath (S–10H, ZEALWAY, Beijing, China). Following this, it was homogenized at a speed of 12,000 r/min for an additional 2 min with the aid of a digital homogenizer (IKA T18 Digital, Staufen, Germany), ultimately resulting in the formation of AEM suspensions. After standing for 24 h at 4 °C, the solution was vacuum filtered and dried at 40 °C for 30 min to obtain AEM.

CH solution was prepared following the method outlined by Jiang et al. [19] with minor adjustments. Initially, CH was dissolved in a 0.5% acetic acid solution (*w*/*w*) to create a 1.5% (*w*/*w*) CH solution. Subsequently, 30% glycerol (*w*/*w* CH) was added as a plasticizer, and 5% Tween 80 (*w*/*w* CH) was included as a surfactant in the solution. The mixture was then stirred for 1 h. To achieve a pH of 4.5, glacial acetic acid was used for adjustment, following which the solution was subjected to vacuum (−0.1 MPa) for 1 h to eliminate air bubbles.

### 2.3. Treatments and Storing of Fresh-Cut Apple

Select apples of uniform color, size, and appearance, free from mechanical damage, and at similar maturity levels. Peel and horizontally slice the apples into fresh-cut apple slices approximately 0.5 cm thick. Randomly divide the apple slices into five groups: one soaked in distilled water for 2 min (control group), another in distilled water containing 5% apple essence microencapsulation (AEM group), a third in slightly acidic electrolyzed water (SAEW) solution containing 5% apple essence microencapsulation for 2 min (SAEW–AEM group), a fourth in CH solution containing 5% AEM for 2 min (CH–AEM group), and a fifth, firstly in SAEW solution for 2 min and then in CH–AEM coating solution for 2 min (SAEW–CH–AEM). After natural air drying for 60 min, the five groups of fresh-cut apples were stored at 4 °C for 10 days, with testing every two days. For each sampling point, collect 3 replicates of 5 samples from each treatment.

### 2.4. Respiration Rate, Weight Loss, Firmness, and Ion Leakage Determination

Respiration rate was assessed as described by Jiang et al. [20] with some modifications. Fresh-cut apples were placed in a sealed, specialized fresh-keeping box, and the CO_2_ concentration was meticulously measured every two days using a Dansensor CheckPoint gas meter. The respiration rate was subsequently expressed as the measured CO_2_ concentration.

Weight loss was calculated as the percentage of loss relative to the initial weight recorded on day 0. Firmness was assessed using a texture analyzer (TA. XTPLUS, Stable Micro System, Godalming, UK) equipped with a P/2 probe, which has a diameter of 2 mm.

Ion leakage of the sample was quantified as relative electrical conductivity [20]. Briefly, six apple slices were randomly selected for each treatment. The central portion of each slice was sampled using a 10 mm diameter punch, creating six circular holes. These holes were rinsed three times with distilled water and then incubated with 20 mL of distilled water. The conductivities of the solution before complete cell membrane damage (C1) and after (C2) were measured following incubation at 25 °C for 30 min and subsequent boiling for 30 min using a conductometer (DDS-307A, Electronics Co., Ltd., Shanghai, China). Ion leakage was calculated as (C1/C2) × 100%.

### 2.5. Color and Browning Index Determination

The color change of fresh-cut apples was assessed using a colorimeter (Agera, Hunter Associate Laboratory, Inc., Fairfax, VA, USA), which provided color space coordinates (L*, a*, and b*). Six apple slices were chosen for each treatment. The browning index (BI) was calculated as follows [1]:

(1) $$BI=\frac{100\times \left(x-0.31\right)}{0.172}$$

where $x=\left(a^{*}+1.75L^{*}\right)∕\left(5.645L^{*}+a^{*}-3.012b^{*}\right)$.

### 2.6. Juice Yield, Total Soluble Solids (TSS), pH, and Titratable Acidity (TA) Determination

Three fresh-cut apples from each group were homogenized in a mortar and then centrifuged (Z-326 K, Hermle, Wehingen, Germany, GmbH) at 10,000× *g* for 10 min to obtain a supernatant. The weight of the supernatant was measured, and the percentage of supernatant to the total mass represented the juice yield. The supernatant was utilized directly for measuring pH and TSS using a pH meter (Mettler-Toledo MP 220, Schwerzenbach, Switzerland) and a digital refractometer (RHB-32 model Refractometer, JDSU, Milpitas, CA, USA), respectively. TA was analyzed using an automatic potentiometric titrator (907 GPD Titrino, Metrohm, Herisau, Switzerland) [21].

### 2.7. E-Nose Analysis

The aroma profile of fresh-cut apple samples was analyzed using a portable universal cNose system (Baosheng Industrial Development Co., Ltd., Shanghai, China), following the method described by Zhang et al. [21]. The system consisted of a sample device, a detector unit equipped with an array of 18 distinct metal oxide sensors, and corresponding software for data collection and analysis. Initially, 3.0 g of samples was placed into 10 mL headspace sample vials, which were then heated in a water bath at 25 °C for 30 min. The settings for the E-nose system were configured to have a chamber flow rate of 300 mL/min, an injection flow rate of 100 mL/min, and a measurement duration of 120 s.

### 2.8. Statistical Analysis

Experimental data were subjected to analysis of variance (ANOVA), and significant differences at a level of *p* < 0.05 were determined using Duncan’s multiple range tests in the SPSS 21.0 statistical data analytical software (IBM, Chicago, IL, USA).

## 3. Results and Discussion

### 3.1. Physicochemical Characteristics of Fresh-Cut Apple during Storage

#### 3.1.1. Respiratory Rate

**Figure 1:**
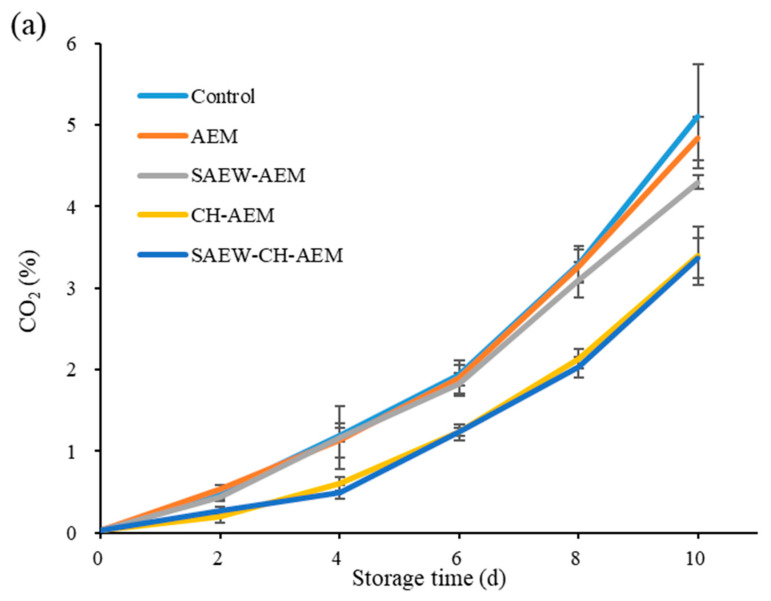

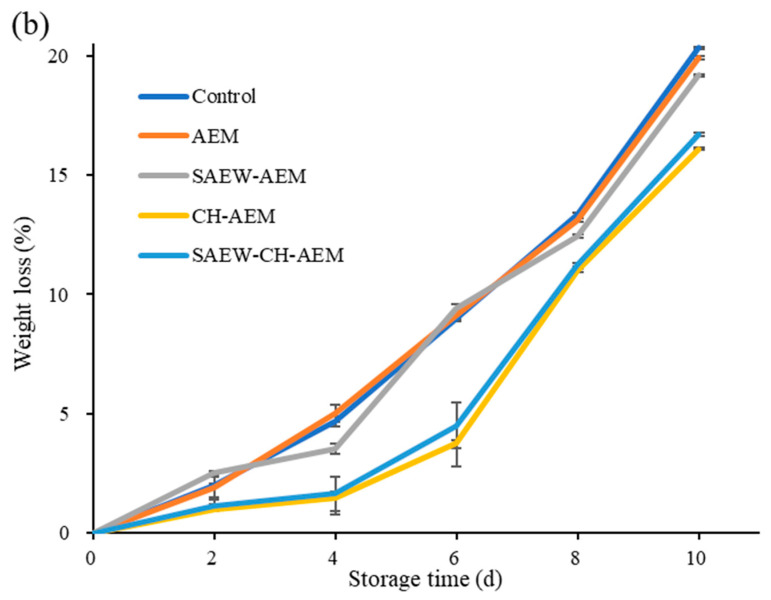
Changes in respiration rate (**a**) and weight loss (**b**) of fresh-cut apple during cold storage. AEM: apple essence microencapsulation, SAEW: slightly acidic electrolyzed water, CH: chitosan.

The respiratory rate, as indicated by the concentration of CO_2_ released, serves as a critical indicator of metabolic activity and senescence in fresh-cut apples. Throughout the storage period, enzymatic browning, microbial proliferation, and physiological changes linked to senescence contributed to an escalation in CO_2_ concentration across all groups [6,20]. Notably, the control group consistently displayed the highest levels of CO_2_ concentration, reaching up to 5.10% after 10 days (Figure 1a), indicating a more rapid respiratory rate compared to the treated groups. In contrast, the SAEW–CH–AEM and CH–AEM groups displayed the most significant reduction in respiratory rate, with final CO₂ concentrations of 3.37% and 3.40%, respectively, representing a reduction of approximately 34% and 33% compared to the control group. This underscores the synergistic effect of CH in mitigating metabolic activity and extending the shelf life of fresh-cut apples. These findings are consistent with those of Yin et al. [22], who noted that coating treatments could reduce the respiration rate by creating a sealed environment, thereby regulating or impeding the exchange between the fruit and its surroundings. Moreover, SAEW demonstrated a partial capability in reducing the respiration rate of fresh-cut apples in the later stages of storage, albeit its synergistic effect with chitosan was less pronounced. This may be attributed to the fact that SAEW effectively suppresses microbial respiration metabolism, thereby reducing the respiration rate of fresh-cut apples to a certain extent [17]. The key data comparison underscores the substantial differences in respiratory rates between the control group and the treated groups, underscoring the effectiveness of the applied treatments in maintaining the quality of fresh-cut apples during storage.

#### 3.1.2. Weight Loss

Due to respiration and transpiration, fresh-cut apples are highly susceptible to water loss, which directly impacts their freshness. Therefore, the weight loss rate serves as a crucial indicator of the quality of fresh-cut fruits [19]. The weight loss data for the apples across the different treatments are presented in Figure 1b. As the storage time increases, there is a consistent increase in weight loss for all treatments. The control group exhibited the highest weight loss, reaching 20.32% after 10 days. Comparatively, the AEM-treated group showed a modest reduction in weight loss, with a final weight loss of 19.92% after 10 days. The AEM group had a slightly lower rate of weight loss compared to the control group. This suggests that the addition of AEM may provide some degree of preservation to the apples, while the effect is not significant compared to the control group. Similarly, the SAEW–AEM-treated group exhibited a slight decrease in weight loss compared to the control group, with a final weight loss of 19.17% after 10 days. In contrast, the CH–AEM-treated group demonstrated a more significant reduction in weight loss, with a final weight loss of 16.10% after 10 days, representing a substantial reduction of 4.22% compared to the control group. Moreover, the SAEW–CH–AEM-treated group demonstrated the most effective preservation, with the lowest weight loss rate of 16.71% after 10 days, representing a reduction of approximately 3.61% compared to the control group. These results suggest that the CH coating is effective in reducing weight loss in fresh-cut apples during storage. Our findings aligned with previous studies that have demonstrated the efficacy of CH-based coatings in reducing weight loss and preserving the quality of fresh-cut fruits [3,9]. CH is known for its film-forming and preservation properties, which help maintain the structural integrity of the fruit and reduce its water loss [3].

#### 3.1.3. Firmness

**Figure 2:**
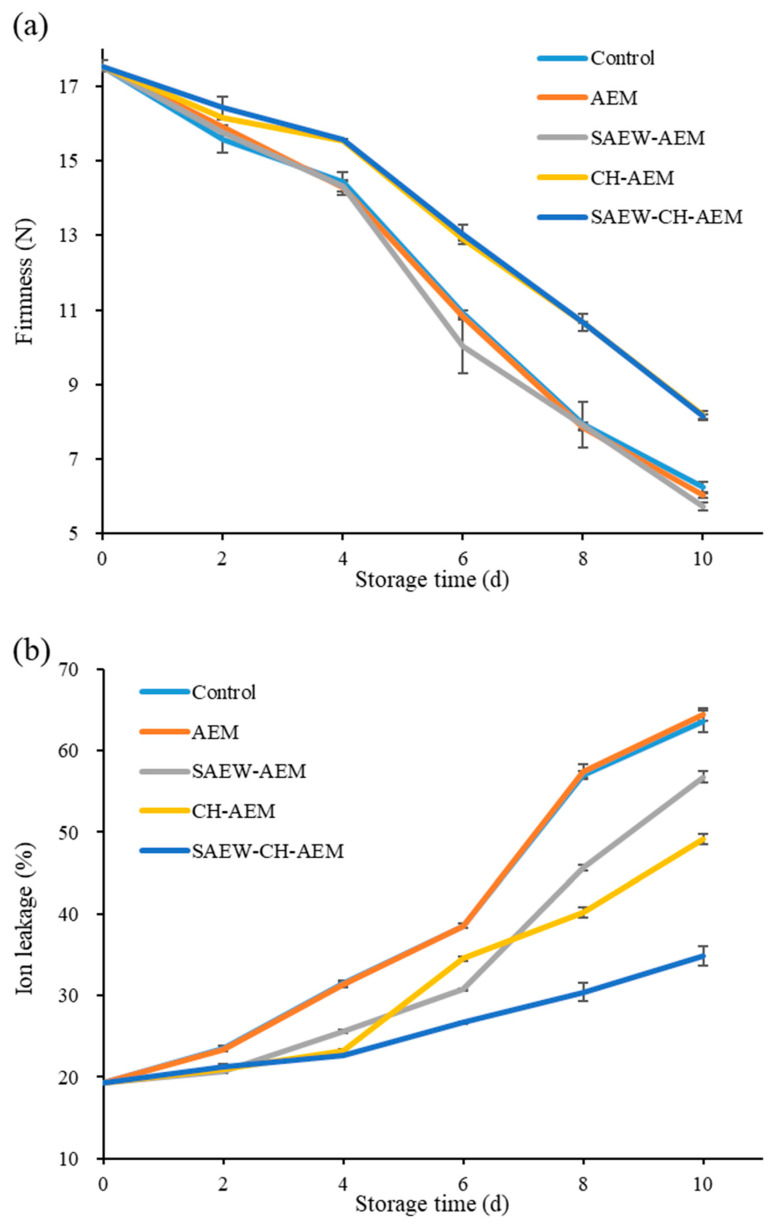
Changes in firmness (**a**) and ion leakage (**b**) of fresh-cut apples during cold storage. AEM: apple essence microencapsulation, SAEW: slightly acidic electrolyzed water, CH: chitosan.

The firmness of apples, a pivotal parameter reflecting fruit quality, exhibited distinctive trends across the various treatment groups during the storage period (Figure 2a). Initial observations unveiled a consistent decline in firmness in the control group (17.54 N to 3.74 N), signifying the inherent degradation process of apples [4,23,24]. The firmness of the AEM and SAEW–AEM groups remained comparable to that of the control group throughout the storage period. Notably, the firmness of the SAEW–AEM group was even lower than that of the control group, declining to 5.73 N after 10 days of storage. These findings suggest that AEM alone may not be adequately effective in substantially extending apple firmness. As expected, AEM’s main function is to enhance apple flavor. That is why we introduced other preservation methods such as SAEM and CH to enhance the shelf life of fresh-cut apples. On the other hand, the CH–AEM and SAEW–CH–AEM combinations exhibited much more promising results. These groups consistently maintained higher firmness values throughout the storage period, with an increase in firmness loss by 30.93%% and 30.45%, respectively, compared to the control group after 10 days of storage. This indicates a more effective approach in preserving apple quality. The similarity in firmness levels between the CH–AEM and SAEW–CH–AEM groups suggests that the CH is a key factor in maintaining apple firmness, while the addition of SAEW might not contribute significantly to this effect. These findings carry significant implications for the fruit industry, as the maintenance of apple firmness is paramount for consumer acceptability and shelf life [25]. These findings suggest that the application of CH, either alone or in combination with SAEW/AEM, effectively preserves the firmness of fresh-cut apples during storage, potentially due to their ability to inhibit enzymatic browning and maintain structural integrity.

#### 3.1.4. Ion Leakage

Ion leakage, serving as an additional indicator of cell membrane integrity, is employed to assess cell membrane damage or viability [26]. It was evaluated throughout the storage period of fresh-cut apples under the various treatment conditions. The control group exhibited a gradual increase in ion leakage over the storage duration, reaching a maximum of 63.59% at the end of the 10-day period (Figure 2b). This rise suggests a progressive deterioration of cell membrane integrity, potentially leading to increased browning and flavor degradation. Similarly, the AEM group showed a pattern of ion leakage comparable to that of the control, with no significant difference observed between the two groups at any time point during the storage period. Specifically, the ion leakage in the AEM group increased from 19.34% to 64.38% over the 10-day storage period, mirroring the trend observed in the control group. This suggests that the AEM may have had a minimal protective effect on cell membrane integrity.

Conversely, the SAEW–AEM group demonstrated a notable reduction in ion leakage compared to both the control and AEM groups. This reduction was particularly evident from day 4 onwards, indicating enhanced preservation of cell membrane integrity attributed to the combined treatment. Ion leakage in the SAEW–AEM group increased to 56.75% over the 10-day storage period, reflecting a significant decrease compared to the control and AEM groups. The antimicrobial and antioxidant properties of SAEW likely contributed to this effect [14]. Moreover, the CH–AEM exhibited a moderate reduction in ion leakage compared to the control and AEM groups, particularly noticeable during the later stages of storage. CH’s film-forming ability and antimicrobial properties likely contributed to improved cell membrane integrity [10], but the effect was less pronounced than with SAEW. Remarkably, the SAEW–CH–AEM group demonstrated the most pronounced reduction in ion leakage throughout the storage period. From day 6 onwards, ion leakage in this group remained significantly lower compared to all other treatment groups, with a final ion leakage of 34.86% after 10 days, representing a substantial reduction of 45.18% compared to the control group. These results suggest that the SAEW–CH–AEM treatment holds promise as an effective strategy for enhancing the preservation of fresh-cut apples by minimizing cell membrane permeability, thereby potentially delaying browning and flavor degradation [10,14].

### 3.2. Color Characteristics of Fresh-Cut Apple during Storage

#### 3.2.1. Sugar Heart

**Figure 3:**
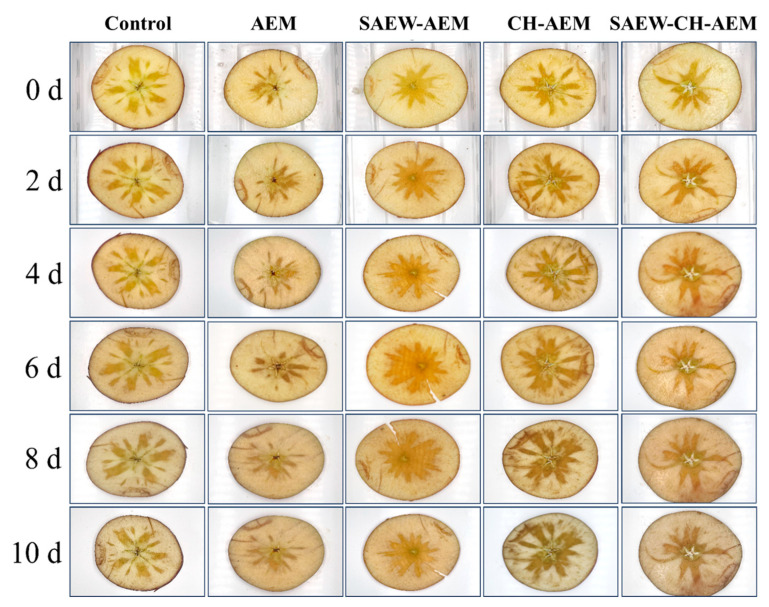
Appearance changes of fresh-cut apples during cold storage. AEM: apple essence microencapsulation, SAEW: slightly acidic electrolyzed water, CH: chitosan.

Over the storage period, the control group showed a gradual decrease in brightness (L*) and an increase in redness (a*) and browning index (BI) (Table S1). This indicates a darkening of the apple surface and an increase in red and brown hues, likely due to natural enzymatic browning processes [1,13]. The AEM treatment initially showed similar trends to the control group, with a decrease in brightness and an increase in redness and BI. However, over time, the rate of change in these indices appeared to be slightly slower compared to the control, suggesting a mitigating effect of AEM on color degradation. Notably, the SAEW–AEM group consistently exhibited lower BI values compared to the control and other treated groups at most storage time points, with a final BI of 57.36 after 10 days, representing a substantial reduction of 13.24% compared to the control group (Table S1). These findings aligned with previous studies, which have also reported the effective inhibition of browning by SAEW through the suppression of PPO enzyme activity [1,14]. The changes in color indicators of fresh-cut apples can also be visually reflected through preservation photos (Figure 3).

The unexpectedly higher browning index observed in the CH–AEM and SAEW–CH–AEM groups compared to the control and other treatment groups suggests that the addition of chitosan may not effectively protect the color of fresh-cut apples. This discrepancy may be attributed to the acidic nature of the chitosan coating solution, which could potentially corrode the apple surface, thereby accelerating enzymatic browning reactions [9]. Under acidic conditions, chitosan coatings may promote the release of metal ions, facilitating reactions with enzymes or polyphenolic compounds present in the apple flesh and exacerbating oxidative processes. Consequently, this chemical reaction could accelerate the browning process, leading to an increase in the browning index. Further research is warranted to optimize chitosan-based coating formulations for fresh-cut apples, considering both their antimicrobial properties and their potential effects on color preservation.

#### 3.2.2. Non-Sugar Heart

Non-sugar heart sections generally exhibited lower L* values and higher a* and b* values compared to sugar heart sections at various storage time points (Table S1). Additionally, BI values in non-sugar heart tended to increase more rapidly, indicating a higher susceptibility to enzymatic browning compared to SH sections, and exhibited more pronounced changes in color attributes. The differences observed between non-sugar heart and sugar heart sections suggest variations in enzymatic browning susceptibility and color retention capabilities. These variations may be attributed to differences in tissue composition, enzymatic activity, and physiological factors between the sugar heart and non-sugar heart regions of the apple [21]. Further research is warranted to elucidate the underlying mechanisms driving these differences and develop targeted strategies to enhance the color preservation of non-sugar heart sections during storage.

### 3.3. Taste Characteristics of Fresh-Cut Apple during Storage

#### 3.3.1. Juice Yield and TSS

**Figure 4:**
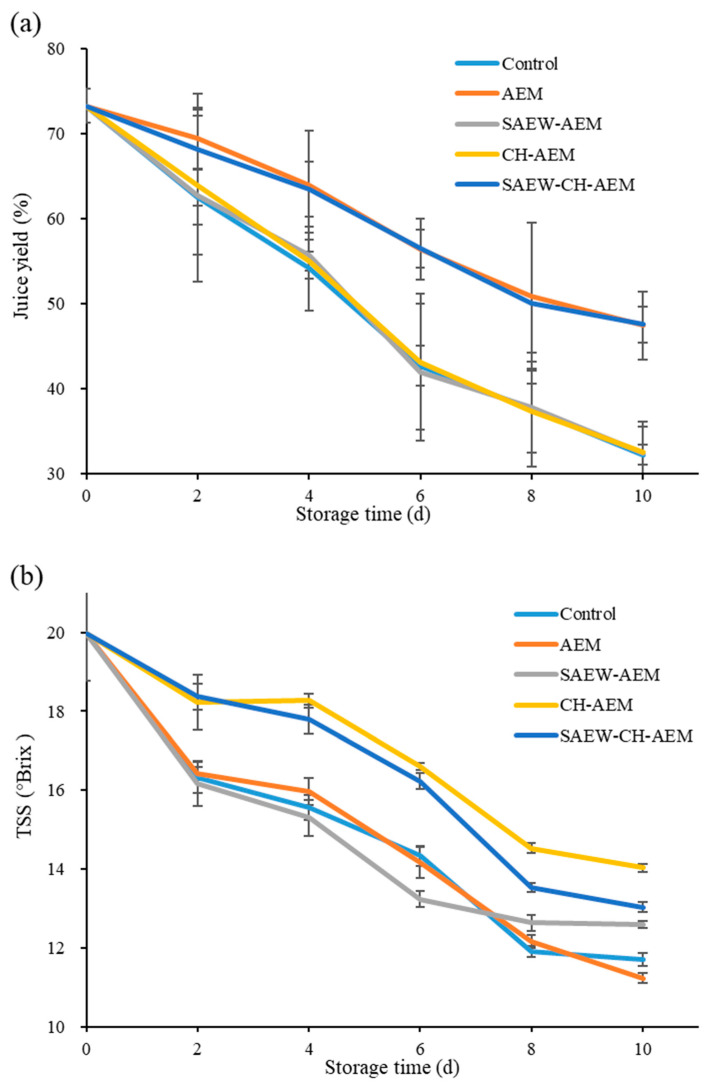
Changes in juice yield (**a**) and TSS (**b**) of fresh-cut apples during cold storage. TSS: total soluble solids, AEM: apple essence microencapsulation, SAEW: slightly acidic electrolyzed water, CH: chitosan.

The variation in juice yield of fresh-cut apples during storage is an important quality indicator directly affecting their taste and appearance. Juice yield in apples primarily depends on the cellular integrity and the water-holding capacity of the fruit tissue. As the storage time progresses, the degradation of cell walls and membranes due to respiration and enzymatic activity leads to increased juicing and a decrease in juice yield. This process is influenced by the rate of respiration, moisture evaporation, and microbial activity [2]. As illustrated in Figure 4a, the juice yield of all groups of fresh-cut apples showed a decreasing trend during storage, with a more pronounced decline observed after day 6. By day 10, the juice yield of the control group had dropped to around 32%. Comparing the changes in juice yield among different treatment groups, it is evident that different treatment methods have varying effects on the juice yield of apples during storage. In the AEM group, the juice yield was relatively higher, reaching 47.43% by day 10, indicating that pure water microencapsulation treatment may help slow down the juicing process of apples. The trends in juice yield change in the SAEW–AEM group and CH–AEM group were similar to that of the AEM group but slightly lower overall, with final juice yields of 32.39% and 32.47%, respectively. This may suggest that the addition of SAEW or CH may assist in controlling apple juicing, but the effect is not as pronounced as when using pure water microcapsules alone. In the SAEW–CH–AEM group, the combined application of SAEW and CH seems to further enhance the control effect on juice yield. Throughout the entire storage period, the juice yield of this group remained consistently high, reaching 47.52% after 10 days, approximately 50% higher than that of the control group. In summary, different treatment methods have varying degrees of impact on the juice yield of apples during storage. Microencapsulation may be an effective method to delay the juicing process of apples, while the individual application of SAEW and chitosan microcapsules shows weaker control effects on juice yield. However, the combined application of SAEW and chitosan microcapsules may have better results, potentially serving as an effective strategy for controlling juice yield during apple storage in the future.

The TSS of apples is a crucial factor reflecting the sweetness of the fruit. A higher TSS value indicates a sweeter fruit [19]. The initial TSS of apple was 16.27 °Brix, which was close to our previous study [21]. It can be seen that in the control group, a gradual decrease in TSS content was observed, declining from an initial value of 16.27 °Brix to 11.93 °Brix at day 10 (Figure 4b). The reduction in TSS highlights the impact of natural aging processes on the sweetness of apples. This decline can be attributed to the evaporation of moisture, respiratory processes, and/or degradation of soluble compounds in apples during storage [4]. Contrastingly, the AEM group displayed a relatively stable TSS trend, exhibiting a gradual increase, particularly in the early storage stages. The TSS values for the AEM group at day 4 were 15.23 °Brix, indicating that AEM contributes to maintaining the sweetness of fresh-cut apples over time. Notably, the CH–AEM group showcased a comparatively higher TSS content, especially in the later storage period. This elevation in TSS further emphasizes the potential synergistic effects of CH and AEM in preserving the sweetness of apples. The combined action of CH and AEM contributes to the enhanced retention of soluble solids. Moreover, the SAEW–CH–AEM group demonstrated a relatively stable TSS content, and the TSS values for the SAEW–CH–AEM group at day 10 were 13.17, suggesting a synergistic enhancement effect of SAEW, CH, and AEM in preserving the TSS content of fresh-cut apples during storage.

#### 3.3.2. pH and TA

**Figure 5:**
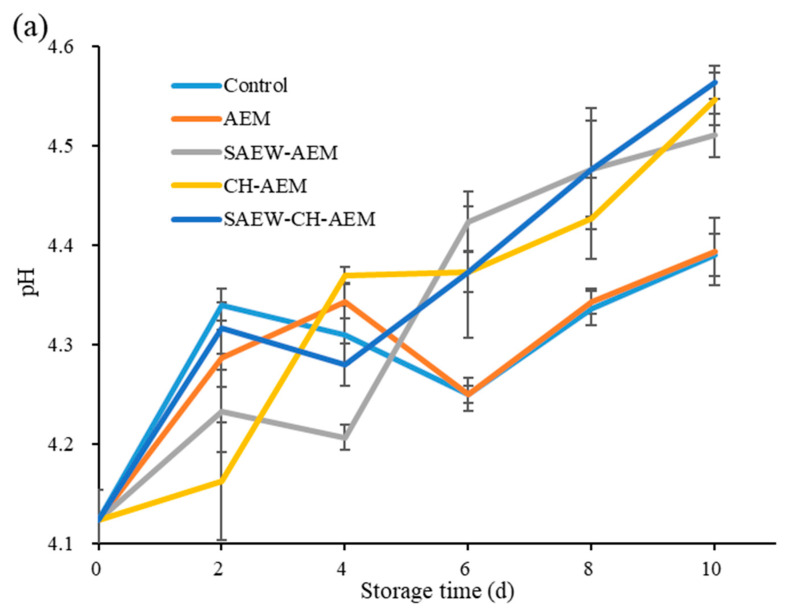

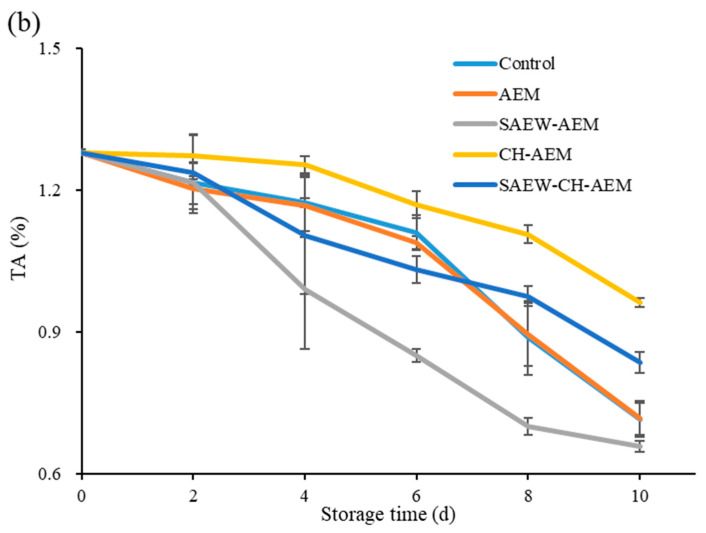
Changes in pH (**a**) and TA (**b**) of fresh-cut apples during cold storage. TA: titratable acidity, AEM: apple essence microencapsulation, SAEW: slightly acidic electrolyzed water, CH: chitosan.

The sourness in fruits and vegetables is primarily attributed to organic acids, including malic acid and acetic acid, which are key components contributing to the excellent flavor of fresh-cut apples [27]. The pH value provides a direct indication of changes in the sensory quality of fresh-cut apples. As illustrated in Figure 5a, with the prolongation of cold storage, the pH values of all samples generally increased, indicating that the acidic substances in fresh-cut apples were gradually consumed by respiration and microbial activity [19]. Over time, the control group’s pH value gradually increased, reaching 4.39 by day 10. The AEM group exhibited a similar trend, suggesting that AEM alone did not significantly alter the pH change pattern compared to the control.

To further characterize the changes in organic acids in apples, we measured the titratable acidity (TA) content of the fruit pulp, as depicted in Figure 5b. With the extension of cold storage, the TA content of all samples exhibited a gradual decrease, primarily attributed to the consumption of organic acids through respiration. The control group showed a steady decline in TA content from an initial 1.28% to 0.71% by day 10. Notably, the TA content in the CH–AEM group was significantly higher than in the other groups. This could be attributed to the fact that chitosan coating more effectively controls the respiratory intensity of fresh-cut apples and inhibits the growth of surface microorganisms, thereby suppressing the consumption of acidic substances within the apples [28]. Conversely, SAEW treatment led to a further decrease in TA content, and the underlying reasons require further investigation.

### 3.4. Aroma Characteristics of Fresh-Cut Apple during Storage

**Figure 6:**
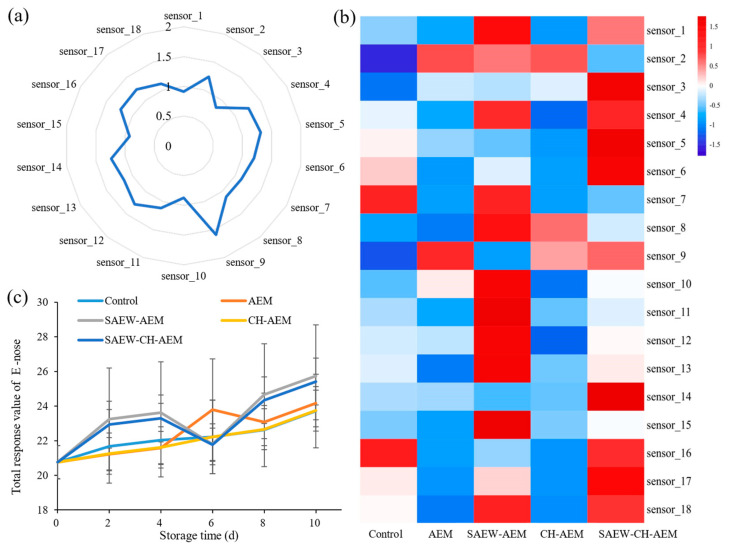
Changes of E-nose response values of fresh-cut apples during cold storage. (**a**) Response value at day 0, (**b**) response value at day 10, and (**c**) total response value variation. AEM: apple essence microencapsulation, SAEW: slightly acidic electrolyzed water, CH: chitosan.

The aroma profiles of different apple fruits were characterized using the E-nose technique. The E-nose is capable of detecting the overall olfactory impression of a mixture without separating individual volatiles [21]. Observing the data from the fresh samples on day 0, we noticed that the response value of Sensor 9 was particularly prominent (Figure 6a), indicating that the initial odor of apples is primarily associated with compounds such as alkanes, alcohols, and ketones [29]. The results are in line with our previous research findings [21]. By the 10th day of storage, significant differences in sensor response values were observed among the apple samples treated by the different methods (Figure 6b), indicating the varied effects of each treatment on the odor of fresh-cut apples.

To comprehensively assess the impact of microencapsulation synergistic treatments on the odor changes of fresh-cut apples, we compiled the response value data from all sensors and conducted a comparative analysis of the trends in total response values (as shown in Figure 6c). In the control group, the response values of the electronic nose steadily increased with storage time, possibly due to the increased production of volatile compounds resulting from respiration and other physiological activities of fresh-cut apples during storage [7,30]. The AEM treatment effectively enhanced the flavor of fresh-cut apples, especially during the mid-storage period. However, the effect of pure AEM treatment did not reach the expected significant level, which may be attributed to the added components of microcapsules and their interactions. The SAEW–AEM treatment group exhibited higher response values in the early stages of storage, possibly due to the significant initial effect of SAEW treatment. As storage time extended, the response values of this treatment group continued to increase, indicating a more significant synergistic effect of SAEW and AEM in the later stages. Notably, the SAEW–AEM group consistently exhibited higher response values compared to the control and other treated groups at most storage time points, with a final response value of 25.74 after 10 days, representing a substantial increase of 8.57% compared to the control group. This treatment not only effectively inhibited the generation of undesirable odors but also enhanced the flavor characteristics of apples to some extent, thus providing consumers with a more enjoyable eating experience. However, it is important to note that an uptick in the electronic nose response value during storage might coincide with the presence of some undesirable odors. Hence, it is imperative for future research to delve deeper into the precise alterations in the aroma of fresh-cut apples, employing sensory evaluation or GC–MS technology.

## 4. Conclusions

This study employed a combination of AEM and CH to prepare a coating solution, which was innovatively combined with SAEW treatment to explore its preservation effects on fresh-cut apples. Experimental results indicated that, compared to the control group, AEM treatment alone significantly enhanced the aroma of fresh-cut apples, improving the sensory quality of the product. However, it did not show significant improvement in other preservation indicators such as surface browning and respiration rate. On the other hand, the application of SAEW significantly delayed the surface browning process of fresh-cut apples, effectively maintaining the appearance quality of the fruit. While the chitosan coating treatment accelerated apple browning to some extent, it effectively inhibited deterioration in quality indicators such as respiration rate and weight loss, demonstrating its unique role in preservation. Combining the above treatment methods, we found that the synergistic treatment of SAEW–CH–AEM exhibited the best performance in preserving fresh-cut apples. Not only did it significantly delay quality deterioration, but it also enhanced the sensory quality of the product, providing an efficient and practical technological means for the preservation of fresh-cut apples. Despite advancements, mechanisms and optimal application methods of combined treatments need clarification. Further research on long-term stability, safety, scalability, and economic feasibility is crucial for practical implementation.

## Data Availability

The original contributions presented in the study are included in the article, further inquiries can be directed to the corresponding author.

## Supplementary Materials

The following supporting information can be downloaded at: https://www.mdpi.com/article/10.3390/foods13101585/s1. Table S1: Color and browning of fresh-cut apples during cold storage.
