# Supplementary material for: Enhancing Fresh-Cut Apple Preservation: Impact of Slightly Acidic Electrolyzed Water and Chitosan–Apple Essence Microencapsulation Coating on Browning and Flavor
Source: Foods. 2024 May 20;13(10):1585. doi: 10.3390/foods13101585 (PMC11121148; doi:10.3390/foods13101585)
Supplement: Supplementary file 1 [file foods-13-01585-s001.zip › foods-3001130-supplementary.pdf]

foods

MDPI

Article

Enhancing Fresh-Cut Apple Preservation: Impact of Slightly Acidic Electrolyzed Water and Chitosan–Apple Essence Microencapsulation Coating on Browning and Flavor

Zhenyu Luo [1,2,3], Guijing Li [1,2,3], Yanlin Du [1,2,3], Junjie Yi [1,2,3], Xiaosong Hu [1,4] and Yongli Jiang [1,2,3,*]

1 Faculty of Food Science and Engineering, Kunming University of Science and Technology, Kunming 650500, China; lzy15708797717@163.com (Z.L.); m13238708120@163.com (G.L.); duyanlin_work@163.com (Y.D.); junjieyi@kust.edu.cn (J.Y.); huxiaos@263.net (X.H.)
2 Key Laboratory of Plateau Characteristic Prepared Food in Yunnan Province, Kunming 650500, China
3 Yunnan Engineering Research Center for Fruit & Vegetable Products, Kunming 650500, China
4 College of Food Science and Nutritional Engineering, China Agricultural University, Beijing 100083, China
* Correspondence: yongli_jiang@kust.edu.cn; Tel.: +86-18817874958

Table S1 Color and browning of fresh-cut apples during cold storage.

		Sugar heart				Non-sugar heart			
Groups	Storage time (d)	L*	a*	b*	BI	L*	a*	b*	BI
Control	0	75.38±0.14a	1.52±0.12a	24.72±0.17a	39.79±0.25a	78.69±0.41a	1.55±0.32a	20.68±0.28a	30.91±0.77a
AEM		75.38±0.14a	1.52±0.12a	24.72±0.17a	39.79±0.25a	78.69±0.41a	1.55±0.32a	20.68±0.28a	30.91±0.77a
SAEM-AEM		75.38±0.14a	1.52±0.12a	24.72±0.17a	39.79±0.25a	78.69±0.41a	1.55±0.32a	20.68±0.28a	30.91±0.77a
CH-AEM		75.38±0.14a	1.52±0.12a	24.72±0.17a	39.79±0.25a	78.69±0.41a	1.55±0.32a	20.68±0.28a	30.91±0.77a
SAEW-CH-AEM		75.38±0.14a	1.52±0.12a	24.72±0.17a	39.79±0.25a	78.69±0.41a	1.55±0.32a	20.68±0.28a	30.91±0.77a
Control	2	73.42±0.24c	1.69±0.18a	27.88±0.04d	47.61±0.44c	75.35±0.14c	2.14±0.45bc	25.71±0.34d	42.33±1.00d
AEM		73.07±0.32c	1.37±0.43a	27.03±0.32c	45.81±0.47b	74.97±1.04c	1.74±0.66b	26.37±0.96d	43.46±0.46d
SAEM-AEM		74.53±0.30b	1.40±0.16a	25.58±0.31b	41.89±0.91a	77.69±0.40b	1.73±0.30b	21.70±0.37b	33.28±0.53b
CH-AEM		72.48±0.23d	1.67±0.12a	29.19±0.40e	51.16±0.56e	67.89±1.65e	3.32±0.68d	27.79±0.75e	54.24±0.83g
SAEW-CH-AEM		72.31±0.09d	1.33±0.18a	28.31±0.23d	49.05±0.67d	73.55±0.32c	2.82±0.21cd	29.63±0.30f	52.38±0.46f
Control	4	71.44±0.22e	2.26±0.14b	30.55±0.27f	55.79±0.54f	73.79±1.07c	2.72±0.11c	27.75±0.08d	48.16±1.14e
AEM		71.46±0.28e	2.83±0.11c	29.96±0.39ef	55.08±0.91f	73.05±0.27c	2.38±0.42c	28.26±0.72d	49.46±1.76e
SAEM-AEM		73.39±0.41d	2.54±0.27b	28.16±0.14d	49.13±0.22d	75.86±0.89b	2.30±0.23c	23.91±0.36c	38.79±0.31c
CH-AEM		69.04±0.74f	2.25±0.18bc	32.26±0.54gh	62.50±1.95h	64.74±0.17f	3.33±0.15de	29.31±0.40f	61.50±1.24i
SAEW-CH-AEM		70.67±0.19e	2.46±0.29bc	31.65±0.20g	59.38±0.99g	70.55±0.18d	2.40±0.47c	30.65±0.10g	57.10±0.57h
Control	6	68.52±0.38f	3.33±0.16d	32.86±0.62h	65.88±2.09hi	68.68±0.69e	3.54±0.20e	29.65±0.43f	58.02±1.29hi
AEM		68.29±0.22f	3.53±0.28d	32.38±0.26h	65.19±0.90h	69.41±0.21e	3.80±0.17e	30.48±0.28g	59.49±0.70i
SAEM-AEM		70.94±0.41e	3.04±0.74d	30.63±0.19f	57.36±0.94f	70.60±0.51d	2.54±0.47c	25.30±0.53d	45.44±1.34d
CH-AEM		58.35±0.15h	3.77±0.14d	31.40±0.33g	77.81±0.85k	59.58±0.03i	2.87±0.22c	31.46±0.22h	74.59±0.72l

SAEW-CH-AEM		61.86±0.67g	3.57±0.23d	30.33±0.13f	68.51±1.55i	65.58±0.32f	3.23±0.37de	31.27±0.12h	65.43±0.46j
Control		61.58±0.27g	3.22±0.05d	33.68±0.38i	78.50±1.63k	61.49±0.35g	2.57±0.22c	30.41±0.25g	68.00±1.34k
AEM		61.59±0.81g	3.49±0.34d	33.40±0.49i	77.97±0.24k	62.02±0.49g	2.85±0.36c	31.47±0.15h	70.60±0.97k
SAEM-AEM	8	67.32±0.22f	3.33±0.04d	31.48±0.35g	63.86±0.93h	65.64±0.30f	2.53±0.28c	27.66±0.20d	55.34±0.61g
CH-AEM		54.66±1.04j	4.37±0.22e	32.62±0.25h	90.73±2.16mn	56.46±0.12k	4.14±0.22f	32.19±0.47j	84.71±1.62n
SAEW-CH-AEM		57.66±0.25i	4.31±0.30e	32.69±0.12h	84.19±0.22l	60.19±0.12h	3.71±0.20e	31.54±0.12h	74.89±0.40l
Control		56.42±0.65ij	4.41±0.24e	33.08±0.50hi	88.34±0.56m	57.77±0.84jk	3.43±0.37e	31.39±0.31h	78.40±1.26m
AEM		55.96±1.16ij	4.41±0.13e	32.80±0.76hi	88.36±1.28m	58.39±0.40j	3.03±0.22de	32.15±0.34j	79.19±1.64m
SAEM-AEM	10	62.36±0.65g	3.39±0.33d	32.45±0.25hi	73.65±0.24j	63.60±0.56g	2.95±0.33d	28.91±0.26d	61.39±0.76i
CH-AEM		51.30±0.39k	4.73±0.24e	33.28±0.19i	102.89±1.87o	54.05±0.29l	4.75±0.20f	33.13±0.63k	94.67±1.46o
SAEW-CH-AEM		53.44±0.38j	4.60±0.41e	32.20±0.18h	92.35±1.00n	56.86±0.09k	3.66±0.35e	32.62±0.19j	84.70±0.65n

Data were shown as the mean ± standard deviation (n=3). Different lower-case letters in the same column indicate differences at the $P < 0.05$ level were significant. AEM: apple essence microencapsulation, SAEW: slightly acidic electrolyzed water, CH: chitosan.
